# Chloridotris(4-chloro­benzyl-κ*C*)(triphenyl­arsine oxide-κ*O*)tin(IV)

**DOI:** 10.1107/S1600536809022247

**Published:** 2009-06-24

**Authors:** Nur Hamimah Johari, Kong Mun Lo, Seik Weng Ng

**Affiliations:** a Department of Chemistry, University of Malaya, 50603 Kuala Lumpur, Malaysia

## Abstract

The Sn^IV^ atom in the title compound, [Sn(C_7_H_6_Cl)_3_Cl(C_18_H_15_AsO)], shows a distorted C_3_ClOSn trigonal bipyramidal coordination; the axial O—Sn—Cl angle is 170.22 (4)°.

## Related literature

For the synthesis of tri(4-chloro­benz­yl)tin chloride, see: Sisido *et al.* (1961 ▶). Triphenyl­arsine oxide affords a small number of adducts with triorganotin(IV) Lewis acids; see: Lo *et al.* (2000 ▶); Nardelli *et al.* (1977 ▶); Ng & Kumar Das (1993 ▶).
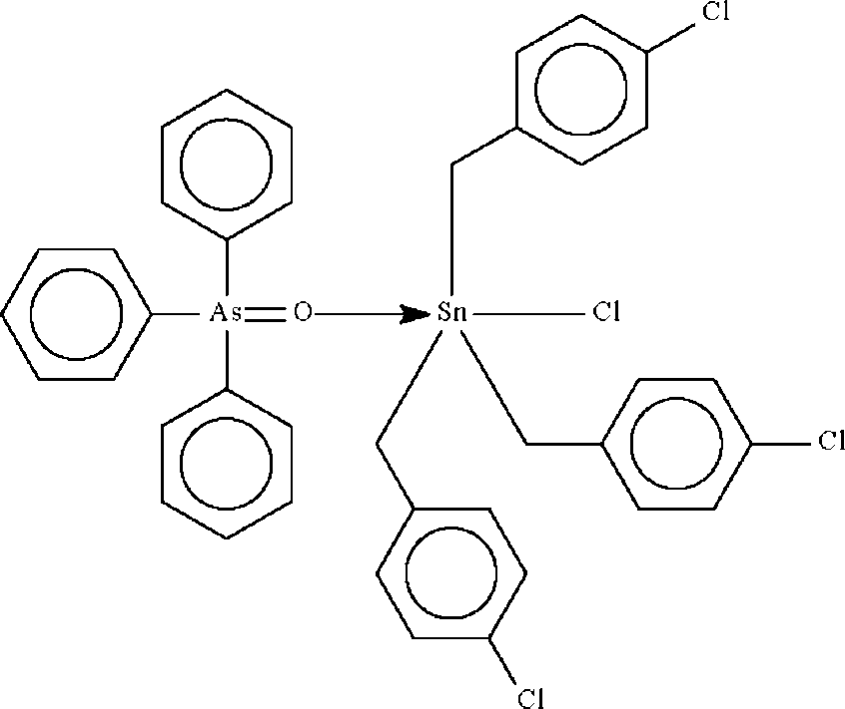


## Experimental

### 

#### Crystal data

[Sn(C_7_H_6_Cl)_3_Cl(C_18_H_15_AsO)]
*M* *_r_* = 853.06
Monoclinic, 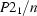

*a* = 17.5639 (2) Å
*b* = 11.0471 (2) Å
*c* = 18.5782 (3) Å
β = 95.818 (1)°
*V* = 3586.2 (1) Å^3^
*Z* = 4
Mo *K*α radiation
μ = 1.96 mm^−1^
*T* = 140 K
0.35 × 0.30 × 0.25 mm

#### Data collection

Bruker SMART APEX diffractometer
Absorption correction: multi-scan (*SADABS*; Sheldrick, 1996 ▶) *T*_min_ = 0.548, *T*_max_ = 0.640
24453 measured reflections
8174 independent reflections
7220 reflections with *I* > 2σ(*I*)
*R*_int_ = 0.018

#### Refinement

*R*[*F* ^2^ > 2σ(*F* ^2^)] = 0.023
*wR*(*F* ^2^) = 0.062
*S* = 1.01
8174 reflections
415 parameters
H-atom parameters constrained
Δρ_max_ = 1.16 e Å^−3^
Δρ_min_ = −0.68 e Å^−3^

### 

Data collection: *APEX2* (Bruker, 2007 ▶); cell refinement: *SAINT* (Bruker, 2007 ▶); data reduction: *SAINT*; program(s) used to solve structure: *SHELXS97* (Sheldrick, 2008 ▶); program(s) used to refine structure: *SHELXL97* (Sheldrick, 2008 ▶); molecular graphics: *X-SEED* (Barbour, 2001 ▶); software used to prepare material for publication: *publCIF* (Westrip, 2009 ▶).

## Supplementary Material

Crystal structure: contains datablocks global, I. DOI: 10.1107/S1600536809022247/tk2475sup1.cif

Structure factors: contains datablocks I. DOI: 10.1107/S1600536809022247/tk2475Isup2.hkl

Additional supplementary materials:  crystallographic information; 3D view; checkCIF report

## Figures and Tables

**Fig. 1.**
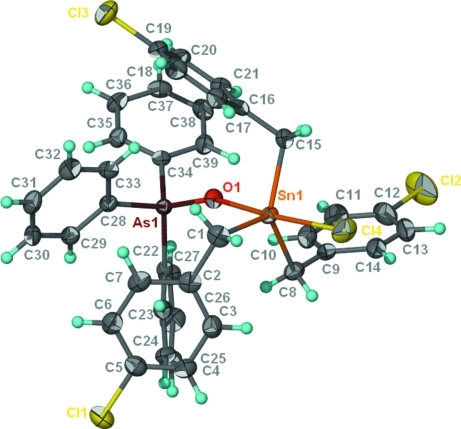
Thermal ellipsoid plot (Barbour, 2001) of (4-ClC7H6)3SnCl.OAs(C6H5)3 drawn at the 70% probability level. Hydrogen atoms are drawn as spheres of arbitrary radius.

## supplementary crystallographic information

### Experimental

Tri(4-chlorobenzyl)tin chloride (0.5 g, 0.93 mmol) and triphenylarsine oxide (0.3 g, 0.93 mmol) were dissolved in chloroform (50 ml).  Slow evaporation of the solvent gave large, well-formed colorless crystals.

### Refinement

Hydrogen atoms were placed at calculated positions (C–H 0.95–0.99 Å) and were treated as riding on their parent atoms with *U*(H) set to 1.2*U*_eq_(C). The final difference Fourier map had a large peak/deep hole in the vicinity of the Sn1 atom.

### Crystal data

**Table:**

[Sn(C_7_H_6_Cl)_3_Cl(C_18_H_15_AsO)]	*F*(000) = 1704
*M**_r_* = 853.06	*D*_x_ = 1.580 Mg m^−^^3^
Monoclinic, *P*2_1_/*n*	Mo *K*α radiation, λ = 0.71073 Å
Hall symbol: -P 2yn	Cell parameters from 9948 reflections
*a* = 17.5639 (2) Å	θ = 2.2–28.3°
*b* = 11.0471 (2) Å	µ = 1.96 mm^−^^1^
*c* = 18.5782 (3) Å	*T* = 140 K
β = 95.818 (1)°	Block, colorless
*V* = 3586.2 (1) Å^3^	0.35 × 0.30 × 0.25 mm
*Z* = 4	

### Data collection

**Table:**

Bruker SMART APEX diffractometer	8174 independent reflections
Radiation source: fine-focus sealed tube	7220 reflections with *I* > 2σ(*I*)
graphite	*R*_int_ = 0.018
ω scans	θ_max_ = 27.5°, θ_min_ = 1.5°
Absorption correction: multi-scan (*SADABS*; Sheldrick, 1996)	*h* = −22→21
*T*_min_ = 0.548, *T*_max_ = 0.640	*k* = −14→14
24453 measured reflections	*l* = −24→24

### Refinement

**Table:**

Refinement on *F*^2^	Primary atom site location: structure-invariant direct methods
Least-squares matrix: full	Secondary atom site location: difference Fourier map
*R*[*F*^2^ > 2σ(*F*^2^)] = 0.023	Hydrogen site location: inferred from neighbouring sites
*wR*(*F*^2^) = 0.062	H-atom parameters constrained
*S* = 1.01	*w* = 1/[σ^2^(*F*_o_^2^) + (0.0322*P*)^2^ + 2.7522*P*] where *P* = (*F*_o_^2^ + 2*F*_c_^2^)/3
8174 reflections	(Δ/σ)_max_ = 0.001
415 parameters	Δρ_max_ = 1.16 e Å^−^^3^
0 restraints	Δρ_min_ = −0.68 e Å^−^^3^

### Fractional atomic coordinates and isotropic or equivalent isotropic displacement parameters (Å^2^)

**Table:**

	*x*	*y*	*z*	*U*_iso_*/*U*_eq_	
Sn1	0.528165 (8)	0.820625 (12)	0.354278 (7)	0.01905 (4)	
As1	0.454087 (11)	0.583674 (17)	0.216927 (10)	0.01551 (5)	
Cl1	0.75643 (4)	0.27072 (6)	0.36668 (4)	0.04362 (15)	
Cl2	0.53088 (6)	1.22894 (8)	0.06049 (5)	0.0676 (3)	
Cl3	0.14901 (4)	0.57827 (6)	0.35680 (4)	0.04224 (15)	
Cl4	0.59378 (3)	0.98287 (6)	0.43764 (3)	0.03751 (14)	
O1	0.46947 (8)	0.70565 (13)	0.26848 (8)	0.0232 (3)	
C1	0.54406 (13)	0.67840 (19)	0.43391 (11)	0.0251 (4)	
H1A	0.5636	0.7150	0.4808	0.030*	
H1B	0.4935	0.6423	0.4399	0.030*	
C2	0.59846 (13)	0.5769 (2)	0.41676 (11)	0.0250 (4)	
C3	0.67409 (13)	0.5993 (2)	0.40221 (11)	0.0271 (5)	
H3	0.6921	0.6803	0.4021	0.032*	
C4	0.72318 (12)	0.5056 (2)	0.38797 (12)	0.0264 (4)	
H4	0.7745	0.5222	0.3793	0.032*	
C5	0.69646 (13)	0.3884 (2)	0.38653 (12)	0.0272 (5)	
C6	0.62260 (13)	0.3621 (2)	0.40032 (12)	0.0274 (5)	
H6	0.6049	0.2808	0.3993	0.033*	
C7	0.57433 (13)	0.4566 (2)	0.41573 (11)	0.0273 (5)	
H7	0.5236	0.4386	0.4258	0.033*	
C8	0.61717 (12)	0.8449 (2)	0.28349 (11)	0.0245 (4)	
H8A	0.6654	0.8672	0.3128	0.029*	
H8B	0.6258	0.7672	0.2590	0.029*	
C9	0.59781 (12)	0.94030 (19)	0.22769 (12)	0.0237 (4)	
C10	0.55992 (13)	0.9105 (2)	0.16050 (12)	0.0290 (5)	
H10	0.5480	0.8282	0.1498	0.035*	
C11	0.53936 (15)	0.9984 (2)	0.10906 (13)	0.0370 (6)	
H11	0.5127	0.9771	0.0638	0.044*	
C12	0.55827 (15)	1.1175 (2)	0.12457 (15)	0.0384 (6)	
C13	0.59802 (15)	1.1498 (2)	0.18935 (15)	0.0379 (6)	
H13	0.6125	1.2316	0.1986	0.045*	
C14	0.61650 (13)	1.0615 (2)	0.24074 (13)	0.0301 (5)	
H14	0.6426	1.0839	0.2861	0.036*	
C15	0.41990 (12)	0.91348 (19)	0.35346 (12)	0.0248 (4)	
H15A	0.4217	0.9674	0.3962	0.030*	
H15B	0.4124	0.9649	0.3097	0.030*	
C16	0.35285 (11)	0.83011 (18)	0.35436 (11)	0.0209 (4)	
C17	0.33463 (12)	0.7764 (2)	0.41844 (11)	0.0251 (4)	
H17	0.3659	0.7925	0.4622	0.030*	
C18	0.27199 (13)	0.7000 (2)	0.41991 (12)	0.0277 (5)	
H18	0.2600	0.6654	0.4642	0.033*	
C19	0.22742 (12)	0.67529 (19)	0.35625 (13)	0.0266 (5)	
C20	0.24387 (12)	0.7258 (2)	0.29136 (12)	0.0259 (4)	
H20	0.2131	0.7075	0.2477	0.031*	
C21	0.30588 (12)	0.80338 (19)	0.29101 (11)	0.0231 (4)	
H21	0.3167	0.8392	0.2467	0.028*	
C22	0.54389 (11)	0.54327 (17)	0.17158 (10)	0.0181 (4)	
C23	0.60824 (12)	0.50616 (19)	0.21602 (11)	0.0223 (4)	
H23	0.6059	0.4977	0.2666	0.027*	
C24	0.67555 (12)	0.4817 (2)	0.18610 (12)	0.0260 (4)	
H24	0.7196	0.4562	0.2161	0.031*	
C25	0.67849 (13)	0.4944 (2)	0.11210 (13)	0.0304 (5)	
H25	0.7247	0.4773	0.0915	0.036*	
C26	0.61465 (14)	0.5319 (2)	0.06813 (12)	0.0333 (5)	
H26	0.6173	0.5412	0.0176	0.040*	
C27	0.54664 (12)	0.5560 (2)	0.09760 (11)	0.0257 (4)	
H27	0.5025	0.5810	0.0674	0.031*	
C28	0.42859 (11)	0.44932 (18)	0.27411 (10)	0.0182 (4)	
C29	0.45851 (12)	0.33508 (18)	0.26293 (11)	0.0222 (4)	
H29	0.4878	0.3211	0.2235	0.027*	
C30	0.44512 (12)	0.24176 (19)	0.31007 (12)	0.0263 (4)	
H30	0.4663	0.1639	0.3036	0.032*	
C31	0.40078 (13)	0.2620 (2)	0.36661 (12)	0.0273 (5)	
H31	0.3919	0.1980	0.3989	0.033*	
C32	0.36943 (13)	0.3753 (2)	0.37621 (11)	0.0273 (5)	
H32	0.3381	0.3881	0.4143	0.033*	
C33	0.38362 (11)	0.47015 (19)	0.33051 (11)	0.0224 (4)	
H33	0.3630	0.5482	0.3376	0.027*	
C34	0.37257 (11)	0.61817 (18)	0.14396 (10)	0.0177 (4)	
C35	0.31898 (12)	0.53077 (19)	0.12201 (11)	0.0222 (4)	
H35	0.3221	0.4524	0.1432	0.027*	
C36	0.26039 (12)	0.5586 (2)	0.06862 (11)	0.0251 (4)	
H36	0.2230	0.4994	0.0532	0.030*	
C37	0.25667 (12)	0.6729 (2)	0.03795 (11)	0.0249 (4)	
H37	0.2167	0.6915	0.0013	0.030*	
C38	0.31019 (13)	0.7599 (2)	0.05986 (12)	0.0271 (5)	
H38	0.3071	0.8379	0.0382	0.033*	
C39	0.36853 (12)	0.73361 (19)	0.11349 (11)	0.0244 (4)	
H39	0.4053	0.7935	0.1293	0.029*	

### Atomic displacement parameters (Å^2^)

**Table:**

	*U*^11^	*U*^22^	*U*^33^	*U*^12^	*U*^13^	*U*^23^
Sn1	0.01672 (7)	0.02216 (7)	0.01808 (7)	−0.00160 (5)	0.00075 (5)	−0.00196 (5)
As1	0.01499 (10)	0.01635 (9)	0.01506 (9)	0.00023 (7)	0.00087 (7)	−0.00054 (7)
Cl1	0.0367 (3)	0.0308 (3)	0.0643 (4)	0.0084 (3)	0.0100 (3)	−0.0015 (3)
Cl2	0.0820 (6)	0.0479 (4)	0.0774 (6)	0.0187 (4)	0.0303 (5)	0.0393 (4)
Cl3	0.0293 (3)	0.0391 (3)	0.0582 (4)	−0.0119 (3)	0.0035 (3)	0.0056 (3)
Cl4	0.0298 (3)	0.0443 (3)	0.0368 (3)	−0.0077 (2)	−0.0044 (2)	−0.0183 (3)
O1	0.0232 (7)	0.0220 (7)	0.0240 (7)	−0.0009 (6)	0.0006 (6)	−0.0071 (6)
C1	0.0311 (12)	0.0295 (11)	0.0144 (9)	0.0038 (9)	0.0004 (8)	0.0053 (8)
C2	0.0272 (11)	0.0312 (11)	0.0158 (9)	0.0025 (9)	−0.0021 (8)	0.0031 (8)
C3	0.0295 (12)	0.0247 (11)	0.0258 (10)	−0.0020 (9)	−0.0038 (9)	−0.0006 (8)
C4	0.0205 (10)	0.0320 (11)	0.0260 (10)	−0.0003 (9)	−0.0011 (8)	0.0000 (9)
C5	0.0276 (11)	0.0277 (11)	0.0256 (10)	0.0044 (9)	−0.0001 (9)	0.0018 (8)
C6	0.0275 (11)	0.0275 (11)	0.0264 (10)	−0.0018 (9)	−0.0023 (9)	0.0052 (9)
C7	0.0232 (11)	0.0344 (12)	0.0237 (10)	−0.0020 (9)	−0.0003 (8)	0.0073 (9)
C8	0.0197 (10)	0.0277 (11)	0.0266 (10)	−0.0019 (8)	0.0055 (8)	−0.0004 (8)
C9	0.0179 (10)	0.0230 (10)	0.0317 (11)	−0.0010 (8)	0.0097 (8)	−0.0013 (8)
C10	0.0322 (12)	0.0238 (11)	0.0316 (11)	−0.0040 (9)	0.0054 (9)	0.0018 (9)
C11	0.0437 (15)	0.0361 (13)	0.0319 (12)	−0.0014 (11)	0.0064 (11)	0.0078 (10)
C12	0.0399 (14)	0.0286 (12)	0.0498 (15)	0.0084 (11)	0.0203 (12)	0.0170 (11)
C13	0.0378 (14)	0.0203 (11)	0.0592 (16)	−0.0010 (10)	0.0228 (12)	−0.0026 (11)
C14	0.0268 (11)	0.0250 (11)	0.0404 (13)	−0.0023 (9)	0.0137 (10)	−0.0080 (9)
C15	0.0245 (11)	0.0195 (10)	0.0303 (11)	−0.0006 (8)	0.0015 (9)	−0.0032 (8)
C16	0.0187 (10)	0.0175 (9)	0.0264 (10)	0.0045 (7)	0.0027 (8)	−0.0040 (8)
C17	0.0265 (11)	0.0254 (10)	0.0227 (10)	0.0025 (9)	−0.0010 (8)	−0.0038 (8)
C18	0.0282 (11)	0.0289 (11)	0.0270 (11)	0.0016 (9)	0.0071 (9)	0.0026 (9)
C19	0.0169 (10)	0.0241 (10)	0.0388 (12)	−0.0007 (8)	0.0036 (9)	0.0000 (9)
C20	0.0189 (10)	0.0298 (11)	0.0280 (10)	0.0023 (8)	−0.0028 (8)	−0.0021 (9)
C21	0.0209 (10)	0.0253 (10)	0.0230 (10)	0.0038 (8)	0.0014 (8)	0.0014 (8)
C22	0.0164 (9)	0.0173 (9)	0.0211 (9)	−0.0013 (7)	0.0040 (7)	−0.0021 (7)
C23	0.0217 (10)	0.0225 (10)	0.0226 (10)	0.0001 (8)	0.0022 (8)	0.0004 (8)
C24	0.0163 (10)	0.0279 (11)	0.0338 (11)	0.0003 (8)	0.0028 (8)	−0.0041 (9)
C25	0.0227 (11)	0.0334 (12)	0.0368 (12)	−0.0017 (9)	0.0116 (9)	−0.0115 (10)
C26	0.0353 (13)	0.0427 (14)	0.0233 (11)	−0.0032 (11)	0.0102 (9)	−0.0057 (10)
C27	0.0244 (11)	0.0319 (11)	0.0207 (10)	0.0004 (9)	0.0018 (8)	−0.0029 (8)
C28	0.0167 (9)	0.0202 (9)	0.0171 (9)	−0.0002 (7)	−0.0005 (7)	0.0020 (7)
C29	0.0204 (10)	0.0224 (10)	0.0240 (10)	−0.0007 (8)	0.0035 (8)	−0.0022 (8)
C30	0.0251 (11)	0.0176 (10)	0.0354 (11)	−0.0024 (8)	0.0003 (9)	0.0012 (8)
C31	0.0296 (12)	0.0251 (11)	0.0266 (11)	−0.0071 (9)	0.0000 (9)	0.0059 (8)
C32	0.0270 (11)	0.0326 (12)	0.0232 (10)	−0.0026 (9)	0.0062 (9)	0.0029 (9)
C33	0.0202 (10)	0.0242 (10)	0.0231 (10)	0.0027 (8)	0.0034 (8)	0.0011 (8)
C34	0.0153 (9)	0.0213 (10)	0.0164 (9)	0.0020 (7)	0.0019 (7)	−0.0009 (7)
C35	0.0232 (10)	0.0212 (10)	0.0219 (10)	−0.0033 (8)	0.0011 (8)	0.0033 (8)
C36	0.0200 (10)	0.0315 (11)	0.0231 (10)	−0.0059 (8)	−0.0009 (8)	0.0004 (8)
C37	0.0204 (10)	0.0345 (12)	0.0190 (9)	0.0055 (9)	−0.0012 (8)	0.0017 (8)
C38	0.0305 (12)	0.0233 (10)	0.0270 (11)	0.0046 (9)	0.0002 (9)	0.0072 (8)
C39	0.0235 (10)	0.0211 (10)	0.0279 (11)	−0.0020 (8)	−0.0002 (8)	0.0009 (8)

### Geometric parameters (Å, °)

**Table:**

Sn1—C1	2.157 (2)	C16—C21	1.399 (3)
Sn1—C8	2.159 (2)	C17—C18	1.389 (3)
Sn1—C15	2.159 (2)	C17—H17	0.9500
Sn1—O1	2.210 (1)	C18—C19	1.378 (3)
Sn1—Cl4	2.5646 (5)	C18—H18	0.9500
As1—O1	1.6594 (14)	C19—C20	1.385 (3)
As1—C28	1.9049 (19)	C20—C21	1.387 (3)
As1—C34	1.9076 (18)	C20—H20	0.9500
As1—C22	1.915 (2)	C21—H21	0.9500
Cl1—C5	1.736 (2)	C22—C27	1.387 (3)
Cl2—C12	1.746 (2)	C22—C23	1.392 (3)
Cl3—C19	1.746 (2)	C23—C24	1.383 (3)
C1—C2	1.528 (3)	C23—H23	0.9500
C1—H1A	0.9900	C24—C25	1.388 (3)
C1—H1B	0.9900	C24—H24	0.9500
C2—C7	1.394 (3)	C25—C26	1.382 (3)
C2—C3	1.404 (3)	C25—H25	0.9500
C3—C4	1.389 (3)	C26—C27	1.389 (3)
C3—H3	0.9500	C26—H26	0.9500
C4—C5	1.376 (3)	C27—H27	0.9500
C4—H4	0.9500	C28—C29	1.391 (3)
C5—C6	1.379 (3)	C28—C33	1.394 (3)
C6—C7	1.392 (3)	C29—C30	1.388 (3)
C6—H6	0.9500	C29—H29	0.9500
C7—H7	0.9500	C30—C31	1.388 (3)
C8—C9	1.493 (3)	C30—H30	0.9500
C8—H8A	0.9900	C31—C32	1.386 (3)
C8—H8B	0.9900	C31—H31	0.9500
C9—C14	1.394 (3)	C32—C33	1.387 (3)
C9—C10	1.393 (3)	C32—H32	0.9500
C10—C11	1.384 (3)	C33—H33	0.9500
C10—H10	0.9500	C34—C35	1.381 (3)
C11—C12	1.380 (4)	C34—C39	1.394 (3)
C11—H11	0.9500	C35—C36	1.389 (3)
C12—C13	1.376 (4)	C35—H35	0.9500
C13—C14	1.380 (4)	C36—C37	1.384 (3)
C13—H13	0.9500	C36—H36	0.9500
C14—H14	0.9500	C37—C38	1.377 (3)
C15—C16	1.496 (3)	C37—H37	0.9500
C15—H15A	0.9900	C38—C39	1.386 (3)
C15—H15B	0.9900	C38—H38	0.9500
C16—C17	1.396 (3)	C39—H39	0.9500
			
C1—Sn1—C15	113.80 (8)	C17—C16—C15	121.31 (18)
C1—Sn1—C8	117.18 (8)	C21—C16—C15	121.21 (19)
C15—Sn1—C8	128.92 (8)	C18—C17—C16	121.69 (19)
C1—Sn1—O1	95.43 (7)	C18—C17—H17	119.2
C15—Sn1—O1	85.52 (7)	C16—C17—H17	119.2
C8—Sn1—O1	86.88 (7)	C19—C18—C17	119.1 (2)
C1—Sn1—Cl4	94.35 (6)	C19—C18—H18	120.5
C15—Sn1—Cl4	90.79 (6)	C17—C18—H18	120.5
C8—Sn1—Cl4	88.42 (6)	C18—C19—C20	121.1 (2)
O1—Sn1—Cl4	170.22 (4)	C18—C19—Cl3	119.78 (18)
O1—As1—C28	110.16 (8)	C20—C19—Cl3	119.11 (17)
O1—As1—C34	108.33 (8)	C21—C20—C19	119.10 (19)
C28—As1—C34	110.49 (8)	C21—C20—H20	120.5
O1—As1—C22	110.49 (8)	C19—C20—H20	120.5
C28—As1—C22	108.33 (8)	C20—C21—C16	121.5 (2)
C34—As1—C22	109.05 (8)	C20—C21—H21	119.2
As1—O1—Sn1	156.89 (9)	C16—C21—H21	119.2
C2—C1—Sn1	115.60 (14)	C27—C22—C23	120.65 (19)
C2—C1—H1A	108.4	C27—C22—As1	121.68 (15)
Sn1—C1—H1A	108.4	C23—C22—As1	117.59 (15)
C2—C1—H1B	108.4	C24—C23—C22	119.66 (19)
Sn1—C1—H1B	108.4	C24—C23—H23	120.2
H1A—C1—H1B	107.4	C22—C23—H23	120.2
C7—C2—C3	117.2 (2)	C23—C24—C25	119.8 (2)
C7—C2—C1	120.5 (2)	C23—C24—H24	120.1
C3—C2—C1	122.3 (2)	C25—C24—H24	120.1
C4—C3—C2	121.5 (2)	C26—C25—C24	120.4 (2)
C4—C3—H3	119.2	C26—C25—H25	119.8
C2—C3—H3	119.2	C24—C25—H25	119.8
C5—C4—C3	119.2 (2)	C25—C26—C27	120.2 (2)
C5—C4—H4	120.4	C25—C26—H26	119.9
C3—C4—H4	120.4	C27—C26—H26	119.9
C4—C5—C6	121.3 (2)	C22—C27—C26	119.2 (2)
C4—C5—Cl1	119.70 (18)	C22—C27—H27	120.4
C6—C5—Cl1	118.98 (18)	C26—C27—H27	120.4
C5—C6—C7	119.0 (2)	C29—C28—C33	120.91 (19)
C5—C6—H6	120.5	C29—C28—As1	120.67 (15)
C7—C6—H6	120.5	C33—C28—As1	118.28 (15)
C6—C7—C2	121.8 (2)	C30—C29—C28	119.3 (2)
C6—C7—H7	119.1	C30—C29—H29	120.4
C2—C7—H7	119.1	C28—C29—H29	120.4
C9—C8—Sn1	112.48 (14)	C29—C30—C31	120.1 (2)
C9—C8—H8A	109.1	C29—C30—H30	119.9
Sn1—C8—H8A	109.1	C31—C30—H30	119.9
C9—C8—H8B	109.1	C32—C31—C30	120.3 (2)
Sn1—C8—H8B	109.1	C32—C31—H31	119.9
H8A—C8—H8B	107.8	C30—C31—H31	119.9
C14—C9—C10	117.7 (2)	C31—C32—C33	120.3 (2)
C14—C9—C8	121.6 (2)	C31—C32—H32	119.8
C10—C9—C8	120.63 (19)	C33—C32—H32	119.8
C11—C10—C9	121.3 (2)	C32—C33—C28	119.1 (2)
C11—C10—H10	119.3	C32—C33—H33	120.5
C9—C10—H10	119.3	C28—C33—H33	120.5
C12—C11—C10	119.0 (2)	C35—C34—C39	120.96 (18)
C12—C11—H11	120.5	C35—C34—As1	120.68 (15)
C10—C11—H11	120.5	C39—C34—As1	118.36 (14)
C11—C12—C13	121.3 (2)	C34—C35—C36	119.32 (19)
C11—C12—Cl2	119.1 (2)	C34—C35—H35	120.3
C13—C12—Cl2	119.6 (2)	C36—C35—H35	120.3
C12—C13—C14	119.0 (2)	C37—C36—C35	119.76 (19)
C12—C13—H13	120.5	C37—C36—H36	120.1
C14—C13—H13	120.5	C35—C36—H36	120.1
C13—C14—C9	121.6 (2)	C38—C37—C36	120.86 (19)
C13—C14—H14	119.2	C38—C37—H37	119.6
C9—C14—H14	119.2	C36—C37—H37	119.6
C16—C15—Sn1	113.64 (13)	C37—C38—C39	119.9 (2)
C16—C15—H15A	108.8	C37—C38—H38	120.0
Sn1—C15—H15A	108.8	C39—C38—H38	120.0
C16—C15—H15B	108.8	C38—C39—C34	119.18 (19)
Sn1—C15—H15B	108.8	C38—C39—H39	120.4
H15A—C15—H15B	107.7	C34—C39—H39	120.4
C17—C16—C21	117.47 (19)		
			
C28—As1—O1—Sn1	65.5 (2)	C17—C18—C19—Cl3	179.33 (17)
C34—As1—O1—Sn1	−173.5 (2)	C18—C19—C20—C21	−0.6 (3)
C22—As1—O1—Sn1	−54.1 (2)	Cl3—C19—C20—C21	179.63 (16)
C1—Sn1—O1—As1	−44.1 (2)	C19—C20—C21—C16	1.1 (3)
C15—Sn1—O1—As1	−157.6 (2)	C17—C16—C21—C20	−0.5 (3)
C8—Sn1—O1—As1	72.9 (2)	C15—C16—C21—C20	179.69 (19)
C15—Sn1—C1—C2	154.03 (15)	O1—As1—C22—C27	−110.97 (17)
C8—Sn1—C1—C2	−22.72 (19)	C28—As1—C22—C27	128.28 (17)
O1—Sn1—C1—C2	66.55 (16)	C34—As1—C22—C27	7.98 (19)
Cl4—Sn1—C1—C2	−113.18 (15)	O1—As1—C22—C23	65.81 (17)
Sn1—C1—C2—C7	−125.17 (18)	C28—As1—C22—C23	−54.94 (17)
Sn1—C1—C2—C3	55.5 (2)	C34—As1—C22—C23	−175.24 (15)
C7—C2—C3—C4	−0.5 (3)	C27—C22—C23—C24	−0.1 (3)
C1—C2—C3—C4	178.85 (19)	As1—C22—C23—C24	−176.89 (16)
C2—C3—C4—C5	1.5 (3)	C22—C23—C24—C25	0.1 (3)
C3—C4—C5—C6	−1.3 (3)	C23—C24—C25—C26	0.2 (3)
C3—C4—C5—Cl1	178.47 (16)	C24—C25—C26—C27	−0.6 (4)
C4—C5—C6—C7	0.2 (3)	C23—C22—C27—C26	−0.3 (3)
Cl1—C5—C6—C7	−179.55 (16)	As1—C22—C27—C26	176.38 (17)
C5—C6—C7—C2	0.7 (3)	C25—C26—C27—C22	0.7 (4)
C3—C2—C7—C6	−0.6 (3)	O1—As1—C28—C29	−138.58 (15)
C1—C2—C7—C6	−179.98 (18)	C34—As1—C28—C29	101.77 (17)
C1—Sn1—C8—C9	176.35 (14)	C22—As1—C28—C29	−17.62 (18)
C15—Sn1—C8—C9	0.2 (2)	O1—As1—C28—C33	37.06 (17)
O1—Sn1—C8—C9	81.86 (15)	C34—As1—C28—C33	−82.60 (16)
Cl4—Sn1—C8—C9	−89.56 (14)	C22—As1—C28—C33	158.01 (15)
Sn1—C8—C9—C14	88.9 (2)	C33—C28—C29—C30	−2.0 (3)
Sn1—C8—C9—C10	−90.9 (2)	As1—C28—C29—C30	173.57 (15)
C14—C9—C10—C11	−1.9 (3)	C28—C29—C30—C31	1.5 (3)
C8—C9—C10—C11	177.9 (2)	C29—C30—C31—C32	0.2 (3)
C9—C10—C11—C12	1.2 (4)	C30—C31—C32—C33	−1.6 (3)
C10—C11—C12—C13	1.1 (4)	C31—C32—C33—C28	1.2 (3)
C10—C11—C12—Cl2	−178.8 (2)	C29—C28—C33—C32	0.6 (3)
C11—C12—C13—C14	−2.6 (4)	As1—C28—C33—C32	−175.03 (15)
Cl2—C12—C13—C14	177.29 (19)	O1—As1—C34—C35	−142.08 (16)
C12—C13—C14—C9	1.9 (4)	C28—As1—C34—C35	−21.33 (19)
C10—C9—C14—C13	0.3 (3)	C22—As1—C34—C35	97.62 (18)
C8—C9—C14—C13	−179.5 (2)	O1—As1—C34—C39	37.90 (18)
C1—Sn1—C15—C16	−47.10 (17)	C28—As1—C34—C39	158.65 (16)
C8—Sn1—C15—C16	129.19 (15)	C22—As1—C34—C39	−82.40 (17)
O1—Sn1—C15—C16	46.86 (15)	C39—C34—C35—C36	0.2 (3)
Cl4—Sn1—C15—C16	−142.21 (14)	As1—C34—C35—C36	−179.85 (16)
Sn1—C15—C16—C17	79.8 (2)	C34—C35—C36—C37	0.3 (3)
Sn1—C15—C16—C21	−100.4 (2)	C35—C36—C37—C38	−0.3 (3)
C21—C16—C17—C18	−0.6 (3)	C36—C37—C38—C39	−0.3 (3)
C15—C16—C17—C18	179.2 (2)	C37—C38—C39—C34	0.8 (3)
C16—C17—C18—C19	1.0 (3)	C35—C34—C39—C38	−0.7 (3)
C17—C18—C19—C20	−0.4 (3)	As1—C34—C39—C38	179.31 (17)
